# Immunometabolic factors contributing to obesity-linked hepatocellular carcinoma

**DOI:** 10.3389/fcell.2022.1089124

**Published:** 2023-01-12

**Authors:** May G. Akl, Scott B. Widenmaier

**Affiliations:** ^1^ Department of Anatomy, Physiology, and Pharmacology, University of Saskatchewan, Saskatoon, SK, Canada; ^2^ Department of Physiology, University of Alexandria, Alexandria, Egypt

**Keywords:** immunometabolism, hepatocellular carcinoma (HCC), stress pathways, obesity, NAFLD

## Abstract

Hepatocellular carcinoma (HCC) is a major public health concern that is promoted by obesity and associated liver complications. Onset and progression of HCC in obesity is a multifactorial process involving complex interactions between the metabolic and immune system, in which chronic liver damage resulting from metabolic and inflammatory insults trigger carcinogenesis-promoting gene mutations and tumor metabolism. Moreover, cell growth and proliferation of the cancerous cell, after initiation, requires interactions between various immunological and metabolic pathways that provide stress defense of the cancer cell as well as strategic cell death escape mechanisms. The heterogenic nature of HCC in addition to the various metabolic risk factors underlying HCC development have led researchers to focus on examining metabolic pathways that may contribute to HCC development. In obesity-linked HCC, oncogene-induced modifications and metabolic pathways have been identified to support anabolic demands of the growing HCC cells and combat the concomitant cell stress, coinciding with altered utilization of signaling pathways and metabolic fuels involved in glucose metabolism, macromolecule synthesis, stress defense, and redox homeostasis. In this review, we discuss metabolic insults that can underlie the transition from steatosis to steatohepatitis and from steatohepatitis to HCC as well as aberrantly regulated immunometabolic pathways that enable cancer cells to survive and proliferate in the tumor microenvironment. We also discuss therapeutic modalities targeted at HCC prevention and regression. A full understanding of HCC-associated immunometabolic changes in obesity may contribute to clinical treatments that effectively target cancer metabolism.

## 1 Obesity-linked non-alcoholic fatty liver disease and hepatocellular carcinoma: A global and complex problem

Hepatocellular carcinoma (HCC) is one of the most fatal cancers and there are limited therapeutic options (Diehl and Day, 2017; Anstee et al., 2019; Geh et al., 2021; Sung et al., 2021). According to the World Health Organization, HCC related death amounted to approximately 700,000 in 2018 and is expected to reach 1 million in 2030 (Samant et al., 2021). Several factors cause HCC. Most commonly is chronic viral hepatitis, driven by hepatitis B virus (HBV) and hepatitis C virus (HCV) infection. Other factors include alcoholic liver disease, metabolic liver diseases such as hereditary hemochromatosis and Wilson’s disease (Gunjan et al., 2017; Jayachandran et al., 2020), and exposure to toxins such as aflatoxin B1 (Cao et al., 2022). Of rising concern is the impact of non-alcoholic fatty liver disease (NAFLD), which is expected to become the most common cause (Ascha et al., 2010; Karagozian et al., 2014; Estes et al., 2018a; Estes et al., 2018b; Younossi et al., 2018; Ioannou, 2021a). As NAFLD prevalence has reached 37% globally (Le et al., 2019), there is urgent need for therapeutics that can uncouple mechanisms linking NAFLD to HCC onset as well as delay HCC progression and promote remission. However, such treatment strategies will need to consider the metabolic context, as NAFLD has a strong association with obesity but still approximately 40% of cases are non-obese and 20% are lean (Ha et al., 2022). In obesity, there are immunometabolic alterations (Hotamisligil, 2017; Dyck and Lynch, 2018; Lee et al., 2018) that can impact NAFLD incidence and its progression to HCC, which are likely distinct from pathogenic mechanisms in lean people with NAFLD. Future research is needed to clarify similarities and differences. In this review, we will discuss signatures of NAFLD-induced HCC, obesity-linked stressors that contribute to NAFLD and HCC, immunometabolic rewiring that promote HCC initiation and growth, and agents under development to treat NAFLD and HCC.

### 1.1 Non-alcoholic fatty liver disease, hepatocellular carcinoma, and obesity

NAFLD encompasses a spectrum of fatty liver diseases, with simple steatosis being most common and a relatively benign condition that has a low risk for HCC (Serfaty and Lemoine, 2008). In contrast, liver can progress to a more serious pathology called non-alcoholic steatohepatitis (NASH), which has the added distinguishing characteristic of liver inflammation and fibrosis. NASH accounts for approximately 20% of NAFLD cases and associated fibrosis is a turning point for adverse effects (Ekstedt et al., 2015; Povsic et al., 2019); this link between advanced fibrosis and HCC is also a common feature for HCV and alcoholic fatty liver disease (Marot et al., 2017; Ioannou, 2021b; Shiha et al., 2021). When compared to simple steatosis, annual HCC incidence increases by more than 10-fold in people with NASH and even higher when associated with cirrhosis (Negro, 2020; Grgurevic et al., 2021).

Obesity is an independent risk factor for HCC initiation, progression, and invasion (Nair et al., 2002; Calle and Kaaks, 2004; Gan et al., 2018) and also impacts HCC in people with HCV (Minami et al., 2021) and those with alcoholic fatty liver disease (Chiang and McCullough, 2014). There is interest in determining how these interactions contribute to HCC pathogenesis, progression, and treatment (Joshi-Barve et al., 2015; Ntandja Wandji et al., 2020; Bianco et al., 2021a). Larsson and Wolk (Larsson and Wolk, 2007) found the risk of HCC increase by 17% in overweight people and 89% in people with obesity, indicating severity of metabolic imbalance is related to liver pathology. Several mechanisms have been identified that may link obesity to cancers such as HCC *via* abnormal hormone and cytokine action, exposure to metabolic toxicity, oxidative damage, dysregulated cell cycle, and impaired immunity (Calle and Kaaks, 2004; Diehl and Day, 2017; Dyck and Lynch, 2018; Gan et al., 2018; Anstee et al., 2019; Hou et al., 2020; Peiseler et al., 2022). The common theme is chronic metabolic burdens that arise in obesity drive persistently high levels of stress in hepatocytes and other types of liver cells and alter immune responses of the developing and established tumor (Buck et al., 2017; Diehl and Day, 2017; Hotamisligil, 2017; Dyck and Lynch, 2018).

### 1.2 Genetic, molecular, and metabolic signatures of non-alcoholic fatty liver disease and hepatocellular carcinoma

Similar to many cancers, tumor promoting mutations play a key role. Prevalent small nucleotide polymorphic (SNP) risk alleles have been found for patatin-like phospholipase domain containing 3 (*PNPLA3*), transmembrane 6 superfamily member 2 (*TM6SF2*), membrane bound O-acyltransferase domain containing 7 (*MBOAT7*), glucokinase regulator (*GCKR*), and 17ß-hydroxysteroid dehydrogenase type 13 (*HSD17B1*), and interestingly, the products for these genes impact liver metabolism, which may underlie promotion of NAFLD and HCC (Liu et al., 2014a; Liu et al., 2014b; Singal et al., 2014; Mancina et al., 2016; Thabet et al., 2016; Diehl and Day, 2017; Seko et al., 2017; Seko et al., 2018; Anstee et al., 2019; Tang et al., 2019; Bianco et al., 2021a; Bianco et al., 2021b; Geh et al., 2021). PNPLA3 hydrolyzes triglycerides and retinyl esters, TM6SF2 regulates lipoprotein export, and MBOAT7 is involved in phospholipid remodeling, and these three molecules are now recognized as important biomarkers (Geh et al., 2021). Additionally, a group of recent studies sought to identify common and distinct genetic variants that underlie HCC risk in HBV, HCC, alcoholic fatty liver disease, and NASH (Schulze et al., 2015; Fujimoto et al., 2016; Cancer Genome Atlas Research Network, 2017). This includes large multi-platform analyses across a range of ethnicity, age, and gender. The results reinforce a role for mutated cell cycle regulators (e.g., TP53 and CCND1) and identify factors linked to antioxidant defense (e.g., NFE2L2 and KEAP1), inflammation (e.g., IL6 and STAT3), and lipid metabolism (e.g., APOB and CPS1). Moreover, Govaere et al. (2020) characterized the transcriptional signatures and associated plasma biomarkers that correspond with the progression of steatosis to early and late-stage NASH and liver fibrosis, which led them to identify markers with potential diagnostic and prognostic value and revealing a role for impaired sterol homeostasis.

Metabolomic and lipidomic approaches comparing liver and plasma of patients with steatosis, NASH, and cirrhosis has been utilized to identify biomarkers for diagnosis and prognosis (Aranha et al., 2008; Ferslew et al., 2015; Gorden et al., 2015; Mouzaki et al., 2016; Puri et al., 2018; Masoodi et al., 2021). One finding that emerged from liver lipidomic comparisons is that unesterified (free) cholesterol is elevated in NASH compared to steatosis (Puri et al., 2007; Caballero et al., 2009; Min et al., 2012). Subsequent studies have shown elevated cholesterol can crystalize within hepatocytes and surround cytoplasmic lipid droplets and that this feature distinguishes NASH from simple steatosis for humans and mice (Ioannou et al., 2013; Ioannou et al., 2019). Also, mechanistic studies with rodents and cultured cells as well as epidemiological studies show there is a link with increased dietary cholesterol and reveal elevated free cholesterol in liver is not just a biomarker but a causal factor for NASH pathogenesis (Musso et al., 2013; Ioannou, 2016; Horn et al., 2022). Additionally, hepatic oxidized phospholipid has been identified as a signature distinction and causative factor for NASH (Sun et al., 2020). Development of agents to counteract the accumulation of cholesterol and oxidized phospholipid and alleviate the stress they impose on the liver may have substantial therapeutic value.

### 1.3 Preclinical models to study non-alcoholic fatty liver disease and hepatocellular carcinoma

The study of NASH driven HCC in a clinical setting is ideal but has limitations for deciphering mechanisms of pathogenesis. Complementary mouse models are an invaluable resource for this purpose. That being said, it is recognized there is not any one mouse model system that accurately reflects all aspects of NASH or HCC. Instead, there are multiple models that capture certain elements of the disease. These can be classified into three main categories: diet-induced, carcinogen-induced, and genetic models.

Mice fed high fat diet (HFD) composed of 60% lard-based fat develop hepatic steatosis associated with oxidative stress, insulin resistance and inflammation (Brown et al., 2022). However, most experimental mice strains fed this type of diet rarely progress to severe forms of NASH or develop HCC, unless there is a genetic predisposition (Takahashi et al., 2012; Nakagawa et al., 2014). Green et al. (2022) found the addition of high glucose and fructose water to HFD promoted spontaneous induction of NASH driven HCC after 54 weeks. Similarly, diet high in saturated fat, cholesterol and sucrose promote weight gain, insulin resistance, liver injury and inflammation that is comparable to the human disease phenotype (Machado et al., 2015). While addition of cholesterol and fructose to high fat intake seems key to progression of NASH and development of cancer, the mechanisms underlying disease progression are still under investigation (Ribas et al., 2021). In comparison, the methionine and choline deficient diet (MCD) model is a rapid onset and robust dietary model in which liver develops severe steatohepatitis due to defects in lipid droplet and lipoprotein metabolism, but mice fed this diet lack typical associated features of NASH such as obesity and insulin resistance and thus it has limited utility to understand chronic fatty liver stress that leads to NASH (Haberl et al., 2020; Alshawsh et al., 2022). Alternatively, the Gubra Amylin NASH inducing diet (40% kcal fat (of these 46% are saturated fatty acids), 22% fructose, 10% sucrose, 2% cholesterol) has a phenotypical and transcriptomic resemblance to clinically presented NASH and emerges upon long term diet exposure (Hansen et al., 2020). However, it is not yet known if this diet, on its own, promotes HCC.

Chemical carcinogens are used to cause HCC in mice. Though it is difficult to understand how well this can phenocopy human HCC, it is assumed the underlying mechanism is similar (i.e., ROS induced tumor causing mutation) and the effect is robust and reproducible. Recent studies used carcinogens added to steatosis or NASH diet. Administering a single dose of diethylnitrosamine (DEN) in 1 week old mice is capable of inducing spontaneous HCC (Umemura et al., 2016), and this has also been combined with a high fat, choline deficient diet model that bear partial resemblance to NASH induced HCC (Kishida et al., 2016). Likewise, streptozotocin can induce HCC, but with the absence of weight gain due to insulin deficiency (Fujii et al., 2013). Once a week low dose carbon tetrachloride (CCl_4_) for 24 weeks plus HFD is an efficient model to induce liver fibrosis and HCC with resemblance to the human HCC transcriptome profile (Tsuchida et al., 2018).

Genetically engineered models of HCC are also available. Liver specific PTEN deletion mice is a widely used genetic model of NASH induced HCC, in which the mice develop steatohepatitis and more than half develop HCC by 74–78 weeks of age (Horie et al., 2004). MicroRNA122 (miR-122) is highly enriched in liver and an established suppressor of HCC and a mouse model with liver-deletion of miR-122 locus has been generated to study its antitumor function and potential to generate novel therapies (Nakao et al., 2014). Mice with liver-specific or whole body deletion of NFκB essential modulator (NEMO), peroxisome proliferator activated receptor alpha (PPARα), farnesoid x-activated receptor (FXR), acetyl CoA oxidase (ACOX1) and methionine adenosyltransferase 1A (MAT1A) have also been used as models of NASH driven HCC, but poorly resemble clinically presented NASH (Nakagawa, 2015; Febbraio et al., 2019; Mohs et al., 2021). In contrast, MUP-uPA transgenic mice, a model of transient hepatocyte ER stress, when given a HFD develop a surprisingly similar picture of NASH and more than 78% of HFD-fed MUP-uPA mice develop tumors (Nakagawa et al., 2014). Also, the recently established diet-induced animal model of non-alcoholic fatty liver disease and hepatocellular cancer (DIAMOND) mice have emerged as a model of HCC derived NASH. When fed high fat, high carbohydrate, and 0.1% cholesterol diet plus a fructose-glucose solution, 89% of DIAMOND mice develop HCC between 32 and 52 weeks of diet exposure, and this coincides with steatohepatitis that has similar histological and biochemical features of human NASH as well as similar lipogenic, inflammatory, and proapoptotic signaling profiles (Asgharpour et al., 2016). Altogether, there is a large arsenal of *in vivo* tools for preclinical investigation of NASH and HCC.

## 2 Immunometabolic drivers: Focus on reactive oxygen species and cholesterol

Coordinated interactions between metabolism and immunity control homeostasis. Disrupting this interaction underlie a cluster of obesity-linked diseases, including NAFLD (Hotamisligil, 2017). In obesity, there is increased risk of metabolic insults resulting from insulin resistance, diabetes, gut dysbiosis, abnormal bile acid metabolism, and hepatic lipid accumulation which can trigger maladaptive immunological responses that cause further insult and stimulate fibrosis-promoting actions by stellate cells (Ioannou, 2016; Diehl and Day, 2017; Schwabe et al., 2020; Peiseler et al., 2022). Over time, these insults can cause organelle dysfunction and gene mutations that lead to HCC. It is not clear if one type of insult predominates above all others across all populations, and the mechanistic role of each is incompletely understood. For instance, while excess triglyceride (TG) in liver is linked to steatosis and insulin resistance, mechanistic studies suggest TG in lipid droplets has a relatively benign effect on promoting stress that leads to NASH. Yet, certain fatty acids as well as fatty acid-based products and intermediates can promote or counteract liver stress depending on its chemical nature, subcellular location, and whether it is being used for energy or membrane structure (Gluchowski et al., 2017; Loomba et al., 2021). Likewise, there is still more to understand regarding the relationship between bile acids and microbial metabolic products with NAFLD progression (Chavez-Talavera et al., 2017; Tilg et al., 2022). Here, we focus on the role of hepatic oxidative stress, which is a common feature of most NASH-linked metabolic insults, and the role of hepatic cholesterol excess, which has emerged to be a potential key turning point in triggering chronic liver inflammation.

### 2.1 Non-alcoholic fatty liver disease and oxidative stress

Production of cellular energy [i.e., adenosine triphosphate (ATP)] and building blocks that sustain life processes (i.e., proteins, membrane, DNA, etc.) depend on access to molecular components and energy stored within chemical bonds of absorbed nutrients and related intermediates. To gain such access, cells produce enzymes and place these metabolites in subcellular environments that enable chemical reactivity. A caveat is that this chemical reactivity also poses a risk of producing reactive biproducts and that absorbed food often contains xenobiotics that are more challenging to manage. Overproduction of such agents result in generation of reactive oxygen species (ROS). Hepatocytes and other liver cells counteract this problem by clearing xenobiotics and associated endotoxin when it enters portal circulation from the gut. In obesity, chronic nutrient overload can overburden hepatocyte defenses and in turn promote susceptibility to oxidative stress-induced tumor causing mutations.

Hepatocytes employ defense mechanisms that are capable of scavenging ROS and counteracting their damaging effect on cellular constituents but also must preserve physiological amounts of ROS required for signal transduction and proper cell functions. Physiological levels of ROS has a role in pathways such as proliferation and differentiation, combating pathogens, and plasma membrane repair (Murakami and Motohashi, 2015; Horn and Jaiswal, 2018; Sies and Jones, 2020; Lennicke and Cocheme, 2021). Although the oxidative stress response is pivotal in cell homeostasis, chronic exposure prevents these protective mechanisms from functioning adequately, leading to imbalance between ROS and antioxidant molecule production (Mossenta et al., 2020). The enhanced ROS production will chemically react with and consequently cause DNA damage, lipid peroxidation, and protein misfolding (Sosa et al., 2013; Bartolini et al., 2018). Over time, persistence of these harmful effects, in addition to the failure of the DNA repair mechanisms, can lead to genetic mutations, especially in oncogenes and tumor suppressor genes (Guichard et al., 2012) and consequently, cancer initiation.

In liver tissue, major sources of ROS are mitochondria, microsome, and peroxisome, which can occur during free fatty acid (FFA) oxidation (Uchida et al., 2020). To prevent excess accumulation, FFA can be stored in TG and secreted in very low-density lipoproteins, incorporated into phospholipid that are then excreted into bile, or alternatively, oxidized to produce energy (Alves-Bezerra and Cohen, 2017). During NAFLD, FFA oxidation in dysfunctional mitochondrial promote ROS accumulation and liver damage. A microsomal enzyme that mediates ω-oxidation of FFA named CYP4A was found to be abundant in plasma of patients with NAFLD, and accumulation of CYP4A11 in HepG2 human hepatoma cells was associated with buildup of ROS derived product malondialdehyde (MDA), superoxide dismutase (SOD), inflammatory cytokines and liver transaminases (Gao et al., 2020). This may suggest ROS accumulation through CYP4A mediated FFA oxidation may promote NASH progression.

### 2.2 Non-alcoholic fatty liver disease and hepatic cholesterol

Cholesterol has unique properties that support membrane homeostasis, but in excess can promote disease. There are several genetic polymorphisms linked to NASH that are also associated with altered cholesterol metabolism (Horn et al., 2022), which has driven attention to the link between cholesterol and HCC development. Over the last 15 years, it has been established that cholesterol accumulation in liver is a major factor driving the progression of steatosis to NASH. An initial finding was free cholesterol accumulates in liver of people with NASH and not in those with simple steatosis (Puri et al., 2007; Caballero et al., 2009; Min et al., 2012). Subsequent experimental studies have demonstrated hepatic cholesterol accumulation triggers NASH (Ioannou et al., 2013; Musso et al., 2013; Savard et al., 2013; Ioannou, 2016; Ioannou et al., 2017; Hansen et al., 2020; Wang et al., 2020; Ribas et al., 2021; Horn et al., 2022). Most of these studies examined dietary cholesterol. However, cholesterol biosynthesis also has an effect, as the genetic induction of the cholesterol synthesis pathway was shown to result in accumulation of liver cholesterol, NASH, and HCC development (Ribas et al., 2021). Moreover, high expression of cholesterol synthesis enzyme 3β-hydroxysteroid-Δ24 reductase (DHCR24) has been found in human HCC liver, whereas inhibiting DHCR24 impeded tumor growth, invasion, and metastasis both in liver cancer cell lines and in Hep3B xenografts (Wu et al., 2020).

The mechanisms by which hepatic cholesterol excess promote NASH and how this contributes to HCC are still under investigation. Elevated liver cholesterol may alter membrane composition to trigger membrane stress and alter lipid rafts (Fu et al., 2012), and it has been shown to induce HCC-promoting and fibrosis associated signaling pathways (Schwabe et al., 2020; Wang et al., 2020; Wang et al., 2022). Another intriguing mechanism is that excess cholesterol is a risk factor for cholesterol crystal formation, which can trigger NASH. Such crystals have been shown to distinguish between steatosis and NASH in humans and mice (Ioannou et al., 2013; Ioannou et al., 2017; Ioannou et al., 2019), coincide with increased cholesterol, and appear to nucleate at lipid droplets and stimulate NLR family pyrin domain containing 3 (NLRP3) inflammasome activity. This may be a critical step, as NLRP3 is known to sense cholesterol crystals and experimental evidence indicates hepatocyte NLRP3 is required and sufficient to drive liver inflammation and fibrosis (Wree et al., 2014a; Wree et al., 2014b). Deciphering strategies to uncouple hepatic cholesterol accumulation from liver stress and inflammasome activation is likely to have important therapeutic value. However, it is unclear why cholesterol accumulates and crystalizes in liver, and whether hepatocytes are capable of re-solubilizing cholesterol crystals after they form.

## 3 Immunometabolic adaptations In hepatocellular carcinoma

Chronic hepatocyte injury drives the initiation of HCC from NASH, and continual exposure to DNA damaging agents and replacement of hepatocytes results in an established tumor (Ringelhan et al., 2018; Hou et al., 2020; Li et al., 2021; Peiseler et al., 2022). While immune responses are capable of identifying and eliminating such cells, altered immune instruction and dysfunction commonly observed in obesity and that can sustain HCC growth has been identified in NASH. In parallel, the tumor acquires altered metabolic needs and defense systems to sustain survival. Here, we discuss alterations in the metabolic demands and adaptations of the tumor as well as NASH-linked alterations to liver immunity that contribute to the onset and progression of HCC.

### 3.1 Hepatocellular carcinoma rely on glucose metabolism

Cancer cells require tremendous amounts of energy to fulfil their metabolic needs (Hoxhaj and Manning, 2020). The most efficient and accessible source of ATP is glucose. Metabolic conversion of glucose to lactate, even in the presence of oxygen, is a distinguishing factor between normal and cancerous cells (Warburg et al., 1927; Lunt and Vander Heiden, 2011) and is referred to as aerobic glycolysis or the Warburg effect. Although this feature has been known for nearly a century the mechanisms and advantages underlying this metabolic shift are still under investigation. In cancer cells, high demand for energy and macromolecules require a rapid but efficient means of ATP production that correspondingly generate a source of carbon atoms such as lactate and pyruvate that serve as substrates for synthesis of products needed for cell anabolism and proliferation (Hoxhaj and Manning, 2020).

Insulin resistance and consequential hyperglycemia commonly arise in obesity (Hotamisligil, 2017; Lee et al., 2018; Loomba et al., 2021), and liver tumors can take advantage of elevated glucose accessibility. Glucose is imported *via* plasma membrane localized glucose transporters. Tumor cancer cells express high levels of glucose transporter 1 (GLUT1), due to enhanced lactate accumulation and efflux as well as the cancer-associated hypoxic microenvironment (Paltoglou and Roberts, 2005; Jiang, 2017). As a result, GLUT1 is rate limiting for glucose import in cancerous tumors, while virtually undetectable in non-tumor and benign tumor tissue (Younes et al., 1995; Cooper et al., 2003; Amann et al., 2009). Overexpression of GLUT1 appears crucial for HCC growth and survival and this enhanced expression was associated with an additional risk of cancer recurrence and worse prognosis in HCC patients after hepatectomy (Chen et al., 2018a). GLUT1 deletion was found to impair HCC proliferation and angiogenesis (Amann et al., 2009). Another glucose importer expressed in HCC, and not in non-cancerous hepatocytes, is the sodium-glucose cotransporter 2 (SGLT2), which in physiological conditions mediates absorption of filtered glucose by renal proximal convoluted tubules. Interestingly, pharmacological agents that inhibit SGLT2 such as Canagliflozin were developed to block renal absorption of glucose and thus treat diabetes. It turns out this agent may also be useful to treat HCC, as Canagliflozin was shown to disrupt SGLT2-expressing liver cancers by reducing glucose uptake, inhibiting glycolytic metabolism, and attenuating proangiogenic activity (Kaji et al., 2018).

The phosphoinositide 3-kinase (PI3K)/AKT pathway plays a major role in regulating glucose metabolism in HCC (Hoxhaj and Manning, 2020). One mechanism is *via* suppressing thioredoxin-interacting protein (TXNIP), which is a tumor suppressor that regulates glucose flux (Parikh et al., 2007; Katturajan et al., 2022). AKT inhibits TXNIP *via* phosphorylation, and this results in increased expression and function of glucose transporters GLUT1 and GLUT4 (Waldhart et al., 2017). This may be critical for HCC progression as downregulation of TXNIP and its impact on glucose metabolism has been recognized as a hallmark in prostate, lung, and colorectal cancer (Qu et al., 2018; Tang et al., 2020; Hu et al., 2021), and TXNIP downregulation was evident in HBV-related HCCs and contributed to cancer initiation (Zhang et al., 2021). In addition to regulating TXNIP, the PI3K-AKT pathway can activate glycolytic enzymes through phosphorylation and transcriptional activation (Hoxhaj and Manning, 2020). One example is hexokinase 2 (HK2), which phosphorylates glucose to prevent its efflux, and phosphofructokinase 1 (PFK1), which controls a rate limiting step in glycolysis by catalyzing the conversion of fructose 6 phosphate to fructose 1,6 bisphosphate (Hue and Rider, 1987). HK2 was found to be critical for cancer initiation in mouse and human (Patra et al., 2013; Chen et al., 2014), and its expression level has prognostic value for human breast cancer patients (Sato-Tadano et al., 2013). There is now interest to inhibit HK2 in cancer cells and xenograft models (Zheng et al., 2021). In normal physiology glucokinase (GCK) is the main liver hexokinase, but HK2 expression predominates in HCC and is distinctive to cancerous hepatocytes. Moreover, HK2 depletion was shown to promote cancer cell death and stimulated oxidative phosphorylation (Dewaal et al., 2018), revealing the importance of enhanced HK2 expression and indicating blocking HK2 may have important therapeutic impact for patients with HCC.

### 3.2 Adaptations to oxidative stress that promote hepatocellular carcinoma

While persistently elevated ROS cause HCC-promoting mutations necessary for cancer initiation, adaptations to oxidative stress have also been implicated in cancer cell survival, growth, metastasis, and chemotherapy resistance (Kumari et al., 2018). The master regulator of oxidative stress responses, nuclear factor erythroid 2–related factor 2 (NRF2), appears to play an important role. NRF2 complexes with the ROS-sensor kelch like ECH-associated protein-1 (KEAP1). When ROS levels rise, NRF2 will dissociate from KEAP1 and translocate into the nucleus to drive transcriptional programs that promote cell survival. These functions relate to glutathione metabolism, ROS-detoxification, bile acid and lipid metabolism, inflammation, and autophagy (Kamisako et al., 2014; Chambel et al., 2015; Ahmed et al., 2017; Bartolini et al., 2018; Yamamoto et al., 2018; Li et al., 2020). Such action may protect against HCC initiation but after the tumor has been established it appears HCC cells take advantage of this property for growth and survival. This is supported by the fact that NRF2 gain of function mutations have been associated with HCC (Cancer Genome Atlas Research Network, 2017; Ngo et al., 2017), chronic NRF2 activation causes hepatomegaly (Komatsu et al., 2010; He et al., 2020), and upregulation of NRF2 has been observed as a biomarker for HCC (Zhang et al., 2015; Bartolini et al., 2018; Mohs et al., 2021).

Targeting oxidative stress in NASH to prevent HCC is a promising venue. Using antioxidants like vitamin E and C as well as enhancing internal antioxidant defenses may provide resistance to liver injury (Ramos-Tovar and Muriel, 2020). Moreover, targeting NRF2 has been shown to counteract liver toxicity (Camer et al., 2015; Chan et al., 2021; Mohs et al., 2021). Opposingly, enhancing hepatocyte ROS toxicity and promoting ROS induced cancer cell death has been a focus (Wei et al., 2019; Kim et al., 2021). This is either by increasing ROS production or inhibiting antioxidant defense mechanisms (Nogueira and Hay, 2013). For example, use of a glutathione inhibitor augmented the anti-tumor effect of multi-kinase inhibitors on liver cancer cells, an effect potentially driven by augmenting ROS toxicity (Cucarull et al., 2021). A plant-based alkaloid with anti-cancer effects named Copstin was found to mediate autophagy and mitophagy in Hep3B cells by inhibiting the PI3K/Akt/mTOR pathway, which consequently led to ROS accumulation (Kim et al., 2021), and a synthetic chemical preservative, propyl gallate (PG), has been shown to induce ROS production and cell death in HCC cells (Wei et al., 2019). Hence, when it comes to ROS-targeting therapies for NASH and HCC, disease stage is likely to be an important consideration for the design strategy.

### 3.3 Hepatocellular carcinoma and the of role of autophagy

Autophagy is required by cells to maintain energy balance and homeostasis. During this process, autophagosomes sequester cytosolic content and mediate their transport to autolysosomes where the substances are degraded into basic constituents, such as amino acids, nucleic acids, sugars, and fatty acids (Schneider and Cuervo, 2014). Unselective bulk autophagy is enhanced during metabolic emergencies such as in periods of starvation, allowing cells to use their stored nutrients to fulfil their energy and nutrient needs. During cell stress, a more selective - adaptor mediated - form of autophagy is induced to permit selective recycling of faulty components and organelles (Schneider and Cuervo, 2014; Moscat et al., 2016; Poillet-Perez and White, 2019). Presumably, cancer cells need both types of autophagy to maintain the nutrient requirements of these rapidly growing cells and to remove and recycle abnormal organelles. This process, among others, contributes to the immortalized nature of cancer cells, allowing them to proliferate and survive the ongoing insult-induced stress (Poillet-Perez et al., 2015; Poillet-Perez and White, 2019).

In NAFLD, autophagy appears to have a protective effect against lipid accumulation by enhancing lipid mobilization and lipophagy (Schneider and Cuervo, 2014; Filali-Mouncef et al., 2022). For instance, ERK1/2 stimulation was found to improve liver steatosis *via* Atg7-dependent autophagy in leptin resistant db/db mice (Xiao et al., 2016). Conversely, alterations in autophagic flux as observed by high p62 (an autophagy adaptor protein) level and autophagosome accumulation appear to contribute to HCC, although p62 can also have autophagy independent effects (Taniguchi et al., 2016; Umemura et al., 2016; Denk et al., 2019; Tan et al., 2021). Evidence thus far indicate that autophagy contribute to HCC cancer initiation, progression, and invasion (Chen et al., 2018b), and inhibition of autophagy, either chemically or by Atg7 knockdown, has been shown to suppress hepatocarcinogenesis in the activated RAS signaling HCC murine model (Cho et al., 2021). It may be that autophagy must be fine-tuned to meet the requirement of cellular homeostasis (Marot et al., 2017; Ioannou, 2021b; Shiha et al., 2021), but when initiated, cancer cells can use autophagy pathways to maintain growth, proliferation, and survival.

### 3.4 Role of fatty acids in hepatocellular carcinoma

The liver is a primary organ for lipid metabolism. A balance between lipid uptake and synthesis, on one hand, and lipid transport and catabolism, on the other, is required to prevent lipid overload and hepatocyte damage (Alannan et al., 2020). Under normal condition, hepatocytes derive most fatty acid content from external sources such as adipose tissue lipolysis. But in liver cancer cells, *de novo* lipogenesis is upregulated to address enhanced nutrient needs (Sangineto et al., 2020). Acetyl-CoA is a substrate for the synthesis of glucose, lipid, and protein and there has been interest on its role in cancer cell metabolism (Alannan et al., 2020). For example, Acetyl-CoA carboxylase (ACC) is an enzyme required for acetyl-CoA carboxylation into malonyl-Co A and this activity is rate limiting for lipogenesis. Interestingly, ACC activating mutations have been associated with liver carcinogenesis (Nakagawa et al., 2018) and ACC inhibition can protect against DEN-induced HCC in mice (Lally et al., 2019). Also, lipogenesis has been shown to support HCC survival in glucose deprived conditions, and high ACC alpha expression has prognostic value for patients with HCC (Wang et al., 2016). Another important lipogenic enzyme is fatty acid synthase (FASN), which catalyzes the conversion of acetyl-CoA to fatty acid. The drug metformin has been shown to protect against liver cancer by inhibiting FASN in DEN-induced and AKT-overexpression models of HCC (Bhalla et al., 2012; Zhang et al., 2019). In addition to providing nutritional needs, newly synthesized saturated and monounsaturated fatty acids may protect against oxidative damage associated with metabolism of polyunsaturated fatty acids and as a result protect cancer cells from membrane damage (Rysman et al., 2010).

The role of fatty acid oxidation (FAO) is unclear. FAO is generally suppressed in HCC (Hu et al., 2020), likely since cancer cells are more dependent on aerobic glycolysis for energy needs. Interestingly, overexpression of carnitine/acylcarnitine transporter (CACT), a mitochondrial membrane protein responsible for the transport of acylcarnitine into mitochondrial matrix for oxidation, in a HCC cell line and human HCC tumor tissue has been shown to suppress cancer growth and migration due to increased FAO (Yuan et al., 2021). Growing HCC cells have reduced FAO levels and this may be linked to hypoxic challenges faced by cancer cell in the growing tumor (Eales et al., 2016). However, there may be exceptions, as increased FAO was identified to coincide with catenin beta-1 (CTNNB1)-mutated HCC. In this case, translocated mutant *ß*-catenin activates the *ß*-catenin transcriptional functions in the nucleus to promote cancer formation, which would normally promote cell proliferation and differentiation (Rebouissou et al., 2016; Senni et al., 2019), and this HCC appear to thrive on FAO rather than glycolysis (Senni et al., 2019). The mechanism by which this occurs may be informative for understanding distinct HCC subtypes.

### 3.5 Immunological environment in hepatocellular carcinoma

Liver has a unique structure, with its proximity to portal circulation coming from the gut, which requires a similarly unique immune system. This immune system precludes gut derived microbes and microbial compounds such as lipopolysaccharide from reaching systemic circulation. For that to occur without evoking a systemic immune response, liver immune cells have some degree of immunotolerance (Protzer et al., 2012; Jenne and Kubes, 2013). The liver has approximately 80% of resident macrophage population, called Kupffer cells (KCs), in the body and a large population of natural killer cells (NK cells), natural killer T cells (NKT cells), in addition to resident T lymphocytes that constitute innate immunity. Moreover, liver can recruit infiltrating T lymphocytes and B lymphocytes (Ringelhan et al., 2018). Interestingly, metabolic conditions instruct immunological responses (Buck et al., 2017; Dyck and Lynch, 2018) and a growing body of work shows metabolic context associated with obesity promotes hyperactivation of certain immune responses and defects in other immune responses in liver, which in turn contribute to the pathogenesis and progression of NASH and HCC (Ringelhan et al., 2018; Hou et al., 2020; Peiseler et al., 2022).

There is an incomplete understanding of how immune cell types in liver interact with each other and with hepatocytes to promote or guard against tumor development. This is a complex scenario that is context and timing dependent. That being said, it has been shown in NASH-linked HCC that immunosurveillance is impaired, the proportion of monocyte-derived macrophage versus KCs is increased, cancer promoting inflammation is enhanced, and T cells capable of eliminating the tumor are dysfunctional (Ringelhan et al., 2018; Hou et al., 2020; Li et al., 2021; Barreby et al., 2022). In chronic liver inflammation such as in NASH, KCs are depleted and replaced by monocyte-derived macrophage (Reid et al., 2016; Kazankov et al., 2019). This shift has been shown to underlie insulin resistance as well as liver steatosis, inflammation, and fibrosis through release of inflammatory and non-inflammatory factors (Clementi et al., 2009; Stienstra et al., 2010; Papackova et al., 2012; Han et al., 2013; Morinaga et al., 2015; Morgantini et al., 2019; Ramachandran et al., 2020; Daemen et al., 2021). Cytokines that influence resistance to cancer onset include interleukin 10 and 11, whereas cancer promoting cytokines include tumor necrosis factor alpha and interleukin 6 (Ringelhan et al., 2018; Widjaja et al., 2019; Peiseler et al., 2022). Critical transcriptional effectors of the cytokine signaling cascade are signal transducer and activator of transcription 3 and NFκB (Ringelhan et al., 2018; Taniguchi and Karin, 2018; Hou et al., 2020; Li et al., 2021; Peiseler et al., 2022).

Interestingly, obesity-linked metabolic derangements can alter the transcription profile of liver macrophage even before they develop a pro-inflammatory state (Morgantini et al., 2019; Barreby et al., 2022). Likewise, the function and composition of other hepatic immune cell types can also be altered in this metabolic condition to impact progression of NASH to HCC (Ringelhan et al., 2018; Hou et al., 2020; Li et al., 2021), reinforcing that environment has a major influence on immune cell function (Buck et al., 2017; Dyck and Lynch, 2018). The tumor immune microenvironment in HCC has been under intense investigation, which has revealed T cell exhaustion coincides with cancer and distinct roles for various types of macrophages, T cells, and B cells that depend on whether cells are intra-tumoral or peri-tumoral and associated metabolic landscape such as nutrient and oxygen concentration (Ringelhan et al., 2018; Hou et al., 2020; Li et al., 2021). In mice and humans, NAFLD has been associated with a decrease in CD4^+^ T cells, but not CD8^+^ T cells (Ma et al., 2016). Using mice, this study showed CD4^+^ T cell loss resulted from increased mitochondrial ROS generated *via* fatty acid oxidation, and that blockage of ROS-induced CD4^+^ T cell loss can suppress NAFLD-linked HCC. Additionally, liver in human NASH and a mouse model of choline deficient plus high fat diet-induced NASH were both found to contain elevated levels of activated intrahepatic CD8^+^ T cells and NKT cells (Wolf et al., 2014). In mice, these activated cells were shown to cooperatively trigger liver damage to induce HCC. Interestingly, a subsequent study showed immunoglobulin A (IgA^+^) B cells that accumulate in human NASH and in multiple mouse models of NASH are able to suppress cytotoxic effects of the intrahepatic CD8^+^ T cells on the HCC tumor (Shalapour et al., 2017). Interestingly, tumor repressing effects of these cells were restored by treatment with the promising new therapy PD-L1 blockade. Hence, despite the complexity, a growing understanding of how metabolic state influences immune responses may reveal a novel means of eliminating established NASH-linked HCC tumors.

## 4 Therapeutic interventions for non-alcoholic fatty liver disease and hepatocellular carcinoma

**FIGURE 1:**
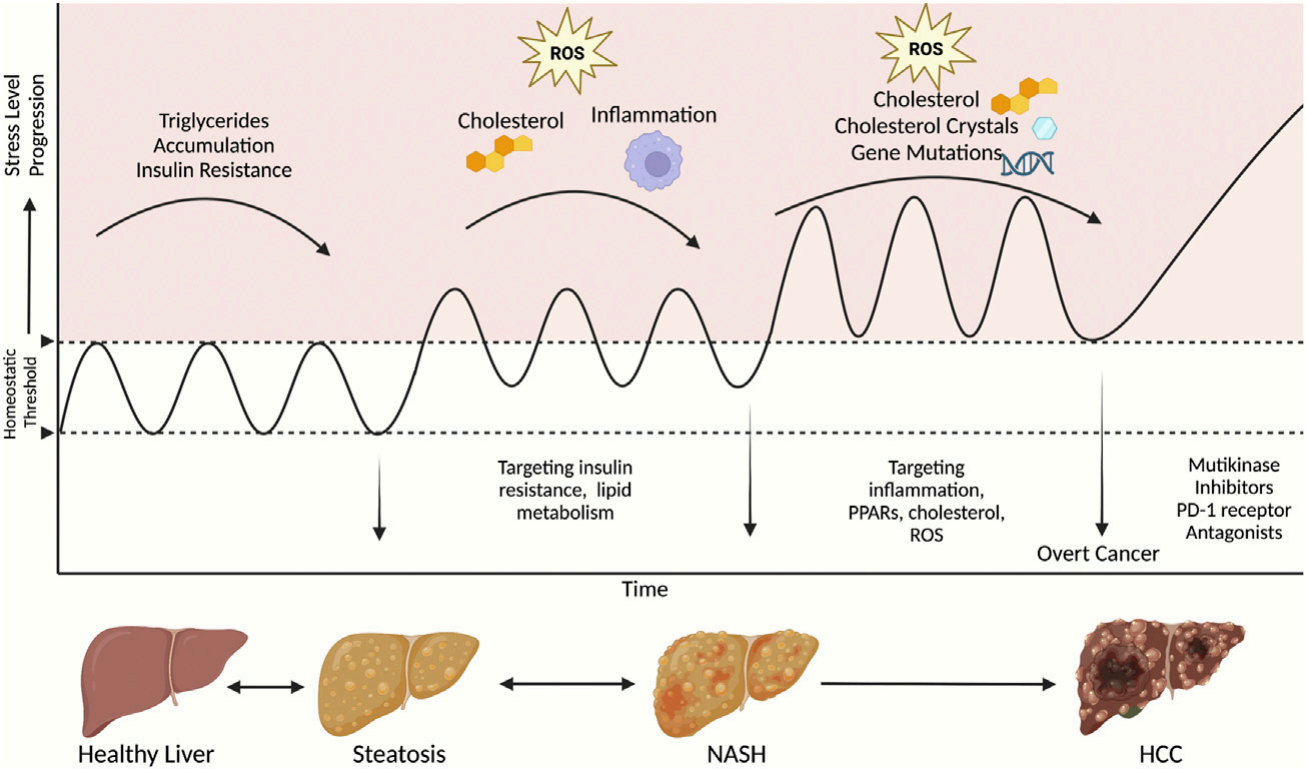
A schematic of therapeutic strategies to target NASH and HCC at different stages of disease progression. This figure was created with Biorender.com.

There is a growing need to prevent HCC and improve outcomes after HCC incidence. To prevent NASH-associated HCC, pharmacological therapies for NASH are under extensive investigations. Treatment of major risk factors of HCC is considered a vital preventive measure. The treatment agents evolve around three components of NASH: steatosis, fibrosis, and inflammation. Although there are no therapies yet approved for NASH, many have shown promising results in preclinical and clinical studies. A summary of available treatments targeting NASH and HCC and how this fit within the stage of disease progression are shown in Figure 1 and Table 1.

**TABLE 1:** Therapeutic interventions for NASH and HCC.

Targets	Class	Drugs	Preclinical NASH	Clinical NASH	Preclinical HCC	Clinical HCC
Insulin resistance	Thiazolidinediones	Pioglitazone Rosiglitazone	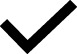 Kalavalapalli et al. (2018); Tahara and Takasu, (2019)	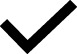 Della Pepa et al. (2021); Panunzi et al. (2021)	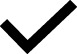 Li et al. (2019)	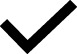 Yang et al. (2015)
	Glucagon like peptide 1based agents	Semaglutide	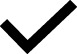 Ding et al. (2006); Valdecantos et al. (2017)	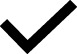 Flint et al. (2021); Newsome et al. (2021)	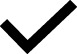 Kojima et al. (2020)	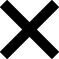
Lipid metabolism	Acetyl-CoA carboxylase inhibitors	Firsocostat	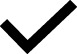 Alkhouri et al. (2020)	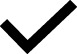 Alkhouri et al. (2020)	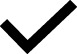	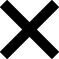
	Stearoyl-coa desaturase 1 inhibitors	Aramchol	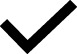 Iruarrizaga-Lejarreta et al. (2017)	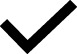 Safadi et al. (2014)	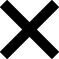	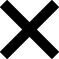
	Thyroid hormone receptor *ß* agonists	Resmetirom	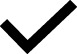 Kannt et al. (2021)	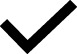 Harrison et al. (2019)	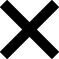	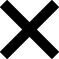
	Statins	Atorvastatin Rouvastatin	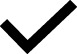	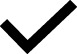 Athyros et al. (2006); Athyros et al. (2010); Kargiotis et al. (2015)	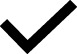	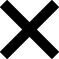
PPARs in liver	PPAR agonists	Lanifibranor	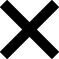 Tong et al. 2019	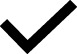 Francque et al. (2021)	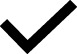 Balandaram et al. (2016); Shen et al. (2020)	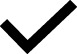
	Fibrates	Fenofibrate	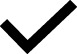 Mahmoudi et al. (2022a); Mahmoudi et al. (2022b)	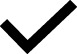 Mahmoudi et al. (2022a)	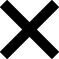	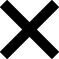
Liver inflammation and fibrosis	CC-chemokine receptors 2 and CC-chemokine receptors 5 antagonists	Cenicriviroc	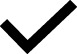 Miura et al. (2012); Lefebvre et al. (2016); Krenkel et al. (2018)	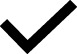 Friedman et al. (2018)	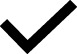 Li et al. (2017)	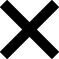
	Galectin 3 inhibitors	Belapectin	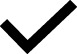 Iacobini et al. (2011)	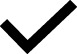 Chalasani et al. (2020)	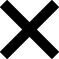	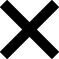
	Farnesoid X receptor agonists	Obeticholic acid Cilofexor Tropifexor	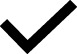 Hernandez et al. (2019); Schwabl et al. (2021); Zhao et al. (2022)	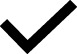 Neuschwander-Tetri et al. (2015); Patel et al. (2020)	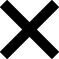	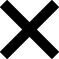
Antiproliferative -Antiangiogenic	Tyrosine kinase inhibitors	Sorafenib Lenvatinib Regorafenib Cabozantinib	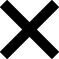	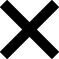	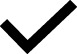	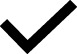 Llovet et al. (2008); Bruix et al. (2017); Kudo et al. (2018); Zhu et al. (2019)
	Programmed death-1 (PD-1) receptor antagonists	Nivolumab	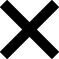	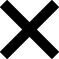	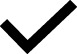	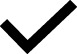 Chiew Woon et al. (2020)
	VEGFR2 antagonists	Ramucirumab	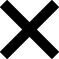	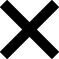	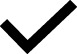	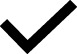 Zhu et al. (2019)

### 4.1 Agents that reduce insulin resistance

Insulin resistance can be a cause and a consequence of hepatocyte lipid accumulation (Loomba et al., 2021). Insulin resistance enhances adipose tissue lipolysis and FFA influx to liver, which consequently stimulate liver lipogenesis and triglyceride accumulation. In contrast, intermediate lipid accumulation in liver can impair hepatocyte insulin signaling and lipid metabolism (Finck, 2018). The thiazolidinediones (TZDs) pioglitazone and rosiglitazone are insulin sensitizing drugs used in the treatment of type II diabetes mellitus. Their action is primarily mediated through peroxisome proliferator-activated receptor-γ (PPARγ) with a resultant enhancement in adipose insulin sensitivity and reduction in free fatty acid transport to liver (Palavicini et al., 2021). TZDs has been shown to improve insulin sensitivity and liver fat accumulation in preclinical animal models of NASH (Kalavalapalli et al., 2018; Tahara and Takasu, 2019) and to be an effective therapy in patients with NASH (Della Pepa et al., 2021; Panunzi et al., 2021). The glucagon like peptide 1 (GLP1) based agent, Semaglutide is another anti-diabetic drug with promising result in clinical trials for NASH (Flint et al., 2021; Newsome et al., 2021). Semaglutide was shown to improve NASH associated steatosis and NASH resolution but did not affect liver fibrosis, which is consistent with studies using preclinical models (Ding et al., 2006; Valdecantos et al., 2017). In addition, TZDs and GLP1 receptor agonists have been shown to reduce hepatocarcinogenesis, cell proliferation and invasion in animal models and cancer cell lines (Yang et al., 2015; Li et al., 2019; Kojima et al., 2020). More clinical trials are underway (Vuppalanchi et al., 2021).

### 4.2 Agents that alter lipid metabolism

Firsocostat is an ACC inhibitor. In liver, Firsocostat decreases steatosis in NASH by inhibiting *de novo* lipogenesis, enhancing lipid oxidation, reducing lipotoxicity, and impairing hepatic stellate cell (HSC) activity (Bates et al., 2020). It has been shown to reduce steatosis and fibrosis markers in preclinical models and clinical trials (Alkhouri et al., 2020). Stearoyl-CoA desaturase 1 (SCD1) is another enzyme involved in fatty acid synthesis that is being targeted to treat NASH. Aramchol is an SCD1 inhibitor used in clinical trials in patients with NASH that was shown to reduce liver fat content in patients after 3 months of daily treatment (Safadi et al., 2014). Other evidence showed Aramchol improved histological markers of NASH associated inflammation and fibrosis in the methionine and choline deficient dietary model of NASH in mice (Iruarrizaga-Lejarreta et al., 2017).

In addition to directly targeting metabolic enzymes, there has been interest in targeting master regulators of lipid metabolic programs. For this purpose, agonists for thyroid hormone receptor *ß* (THRβ) have emerged as a category of drugs for NASH. Activating liver THRβ has been shown to improve lipid profile, bile acid synthesis and lipid oxidation (Sinha et al., 2019). Mice treated with a selective THRβ agonist named Resmetirom have lower liver size, fat content, and improved markers of liver damage and fibrosis (Kannt et al., 2021). Similar fat lowering effect was shown in a recent phase II clinical trial using the same drug (Harrison et al., 2019).

As discussed in Section 2.2 increased hepatic cholesterol, altered cholesterol metabolism, and the precipitation of pro-inflammatory intrahepatic cholesterol crystals are a hallmark of NASH. Thus, counteracting therapeutic agents may alleviate NASH and prevent HCC. Clinical trials investigating the statin class of drugs, which inhibit cholesterol synthesis and reduce circulating low density lipoprotein, support this possibility. Patients in the GREACE study who were treated with atorvastatin showed improved liver function (Athyros et al., 2010), and atorvastatin was found to alleviate NAFLD in a prospective open label study on non-diabetic patients (Athyros et al., 2006), with similar results observed in patients treated with rouvastatin (Kargiotis et al., 2015). However, beneficial effects of statins appear to be ineffective in people with the high-risk allele for PNPLA3 (Dongiovanni et al., 2015), indicating patients may need genotype stratification before a statin can be prescribed to treat NAFLD. Moreover, a recent pre-clinical study showed that a non-statin type of cholesterol-targeting therapy can actually be detrimental for liver health (Ioannou et al., 2022), revealing that the mechanism of drug action will be an important determinant for identifying the most effective therapeutic approach.

### 4.3 Agents that target PPARs in liver

PPARs are nuclear receptors that regulate multiple processes including glucose and lipid metabolism and inflammation. PPAR agonists are under investigation to treat NASH as well as HCC. PPARδ and PPARγ pharmacological and genetic enhancement attenuated hepatic steatosis by inducing autophagy dependent hepatic lipolysis and *ß*-oxidation in mouse livers and primary mouse hepatocytes (Tong et al., 2019) and improving insulin sensitivity (Heikkinen et al., 2007). Lanifibranor is a pan-PPAR agonist in clinical trial for NASH treatment. Patients with active NASH given Lanifibranor showed a significant reduction in steatosis and fibrosis when compared to placebo group and had improved liver enzymes and lipid profile (Francque et al., 2021). PPAR β/δ are also connected with hepatocarcinogenesis and PPAR β/δ agonists show promising results in preclinical models of HCC (Balandaram et al., 2016; Shen et al., 2020). Similar results have been found for the fibrate type drug, fenofibrate (Mahmoudi et al., 2022a; Mahmoudi et al., 2022b). Therefore, modulation of PPAR may turn out to be effective for preventing and for treating HCC.

### 4.4 Agents that target liver inflammation and fibrosis

As a main target of NASH therapeutic agents, inflammatory responses occurring in liver after any chronic injury are currently under investigations, with CC-chemokine receptors (CCRs) serving as a high value target (Lefere et al., 2020). CCR stimulation causes macrophage infiltration and HSC activation (Vuppalanchi et al., 2021), which then is a trigger for pathogenesis of liver fibrosis. HSCs interact with hepatocytes to either maintain normal liver phenotype, promote hepatocyte regeneration in an acute liver injury setting, or promote fibrosis in cases of chronic liver injury and inflammation such as in NASH (Cai et al., 2020). Cenicriviroc is a dual CCR2/CCR5 antagonist that has been investigated extensively in preclinical models of NASH and shown to diminish liver macrophage infiltration, inflammation, and fibrosis (Lefebvre et al., 2016; Krenkel et al., 2018), which is consistent with the effect of deleting CCR2 (Miura et al., 2012). In a randomized clinical trial on patients with NASH, Cenicriviroc reduced fibrosis but not inflammatory scores (Friedman et al., 2018). Moreover, a CCR2 antagonist has been shown to inhibit tumor growth, reduce tumor size and prevent recurrence after resection in a liver cancer experimental model (Li et al., 2017).

Galectin 3 (Gal3) is a ubiquitously expressed protein secreted from macrophages that can activate fibroblasts to induce fibrogenesis *via* transforming growth factor *ß*-dependent pathway (Al Attar et al., 2021). Gal3 deficient mice are protected against NASH (Iacobini et al., 2011). However, an inhibitor of Gal3 called Belapectin was used in patients with NASH and cirrhosis and showed no significant effect on histological scores of fibrosis or on hepatic vein pressure gradient in patients suffering from portal hypertension (Chalasani et al., 2020).

Farnesoid X receptor (FXR) is a bile acid activated nuclear receptor located mainly in liver and intestines, with a regulatory role in lipid and glucose metabolism. FXR may also influence liver fibrosis (Vuppalanchi et al., 2021; Zhao et al., 2022). In a phase II clinical trial, an FXR agonist called Obeticholic acid (OCA) was found to reduce liver fibrosis in 45% of patients with non-cirrhotic NASH (Neuschwander-Tetri et al., 2015). Cilofexor and Tropifexor are also FXR agonists that have demonstrated antifibrotic effect in animal models and are under investigation for their safety in clinical trials (Hernandez et al., 2019; Schwabl et al., 2021; Zhao et al., 2022).

### 4.5 Approved drugs for hepatocellular carcinoma in clinical use

In addition to preventing HCC by ameliorating NASH, there is more agents needed to treat the cancer after onset. In patients with HCC, surgical resection and local ablation are the main lines of treatment in early locally confined tumors. Liver transplantation is a favorable option, especially in cases with microinvasions, as this has a lower rate of recurrence. Locally administrated chemo- and radio-therapeutic drugs are two options provided in advanced non-resectable tumors (Wege et al., 2019; Ayoub et al., 2022). Systemic pharmacological agents that are currently approved for use by the Food and Drug Administration (FDA) are mainly targeting cell proliferation, angiogenesis, and tumor immunity.

The first approved systemic pharmacological option for HCC treatment is Sorafenib, a tyrosine kinase inhibitor with anti-proliferative and anti-angiogenic effect. Sorafenib improved the overall survival rate in patients diagnosed with advanced HCC versus placebo group (Llovet et al., 2008). Several other multi-kinase inhibitors such as Lenvatinib, Regorafenib, and Cabozantinib are now also approved and shown efficacy in improving overall survival (Bruix et al., 2017; Abou-Alfa et al., 2018; Kudo et al., 2018). Another class of agent is Nivolumab, a checkpoint inhibitor that targets programmed death-1 (PD-1) receptor by blocking its natural ligands and consequently activating T cell antitumor capacities and cytokine production (Chiew Woon et al., 2020). Also, Ramucirumab is a human IgG2 monoclonal antibody that inhibits ligand activation of VEGFR2. Ramucirumab has shown improved overall survival with better tolerability in patients with advanced HCC and persistently elevated α-feto protein after sorafenib intake (Zhu et al., 2019).

Overall, the advances in systemic treatment of NASH and HCC, especially those targeting liver cell metabolism add new hope for patients and require more attention in research to improve their efficacy in the prevention of NASH induced HCC development.

## 5 Conclusion

We discuss various immunometabolic factors contributing to NASH and HCC, and how this relates to cancer growth and survival. HCC cells are able to change their metabolism and stress adaptive capacity to fit the new anabolic requirements and tumor microenvironment. Likewise, the immune cell environment is altered in the metabolic state of obesity in a manner that supports tumor growth, and the associated metabolic burdens trigger chronic hepatocyte insults that in turn establish tumor initiation. Hence, in the metabolic context of obesity-linked NASH, strategies to prevent HCC and promote HCC regression require consideration of how these factors differ from other known causes of HCC, such as HBV and aflatoxin B1. There is urgency to acquire this knowledge given the accelerating incidence of obesity-linked fatty liver disease around the world.
